# Supplementary material for: Economic policy uncertainty and common prosperity within the enterprise: Evidence from the Chinese market
Source: PLoS One. 2024 Oct 21;19(10):e0309370. doi: 10.1371/journal.pone.0309370 (PMC11493295; doi:10.1371/journal.pone.0309370)
Supplement: Attachment — Submitted filename: PONE-D-24-11742.docx [file pone.0309370.s002.docx]

**Economic policy uncertainty and common prosperity within the enterprise: Evidence from the Chinese market**

**Reviewer comments**

Utilize theories from economics, finance, and business management to construct a strong theoretical framework.

Explore the impact of EPU on business decisions, investment strategies, and risk appetite.

Discuss theories that are relevant to the concept of common prosperity, such as corporate social responsibility (CSR) and stakeholder theory.

Perform a comprehensive analysis of the current research on EPU and its effects on businesses, with a specific emphasis on the Chinese market.

Please include studies that explore the correlation between EPU and business performance, innovation, and strategic decision-making.

Identify areas of improvement in the existing literature and explain how your study intends to fill those gaps.

Please provide a thorough explanation of the methodological approach, including the techniques used for data collection and analysis.

Provide a rationale for the choice of methods and measurements, using relevant literature to demonstrate their suitability.

Provide clear and transparent explanations of the data sources and collection methods.

Make sure the data accurately reflects the Chinese market or address any limitations that may exist.

Utilise powerful statistical methods and offer comprehensive explanations of the analysis procedure.

Make sure that the selected methods align with the research questions and the characteristics of the data.

Offer a comprehensive analysis of the findings in relation to existing literature and theories.

Examine the potential impact of the findings on businesses and policymakers.

Identify possible constraints and propose opportunities for further investigation.

Take into account the potential for endogeneity in the analysis.

Utilise techniques like instrumental variable (IV) regression or difference-in-differences (DiD) to address potential biases.

Examine the distinct characteristics of the Chinese market and how they might restrict the applicability of the results.

Consider the potential applications of the findings in different contexts or identify areas for further research to delve into this topic further.

Discuss the potential impact of the findings on enterprise management and economic policy in China.

Provide practical recommendations for policymakers and business leaders based on the study's results.
