# Supplementary material for: Economic policy uncertainty and common prosperity within the enterprise: Evidence from the Chinese market
Source: PLoS One. 2024 Oct 21;19(10):e0309370. doi: 10.1371/journal.pone.0309370 (PMC11493295; doi:10.1371/journal.pone.0309370)
Supplement: Attachment — Submitted filename: Response to review comments.docx [file pone.0309370.s003.docx]

Thank you to the editor and reviewers for their valuable comments and suggestions on my paper. I have made significant revisions to the paper based on the reviewers’ feedback and corrected typographical errors (important ones include adjusting the data range to 2011-2020 to exclude the impact of the pandemic, as well as some sentence errors).

Reviewer #1:

1.the description content about the dependent variable enterprise common prosperity is too little, lack of literature support, please add more literature to explain enterprise common prosperity.

Thank you for your valuable comments. We have added a new section in the Theoretical Analysis and Research Hypotheses section, which describes the concept of corporate co-prosperity in detail and cites several relevant literature to support our arguments. The new content has been marked in red for easy review. I hope these additions can enhance the theoretical basis and persuasiveness of this paper. Thank you again for your suggestions.

2.The theoretical analysis part of hypothesis 1 does not reflect how economic uncertainty affects prosperity within firms, so please add a theoretical discussion that clarifies the logic and explains how economic uncertainty affects the shared prosperity of firms.

Thank you for your valuable comments. We have re-examined the basic logic of Hypothesis 1, 2, and 3, and added relevant logic and theoretical discussions after each hypothesis to clarify how economic uncertainty affects the common prosperity of enterprises. We have also added more literature support to enhance the clarity of the logical relationship between variables and the logic of the text discussion. I hope these modifications will improve the theoretical and readability of this article. Thank you again for your suggestions.

3.Hypotheses 2 and 3 also have the same section as above and do not reflect the logical relationship between the variables in terms of the content section, please reorganise the logic and add more literature to clarify the logical relationship between the variables to make the textual discussion more logical.

Thank you for your valuable comments. We have re-examined the basic logic of Hypothesis 1, 2, and 3, and added relevant logic and theoretical discussions after each hypothesis to clarify how economic uncertainty affects the common prosperity of enterprises. We have also added more literature support to enhance the clarity of the logical relationship between variables and the logic of the text discussion. I hope these modifications will improve the theoretical and readability of this article. Thank you again for your suggestions.

4.Please explain specifically how the dependent variable, shared prosperity of firms, is measured, which specific indicators are chosen, what is the measurement methodology, and the reasonableness of the scoring.PleasePleasePlease explainPlease explain.Please explain the use ofPlease explain the rationale for using thisPlease explain the methodology used toPlease explain the methodology used to measurePlease explain the methodology used to measure the enterprisePlease explain the methodology used to measure the common prosperity of enterprises.Please explain the methodology used to measure the shared prosperity of enterprises.Please explain the reasonableness of the methodology used to measure the shared prosperity of firms.Please explain the reasonableness of the methodology used to measure the shared prosperity of enterprises.

Thank you for the reviewer's valuable comments. We have explained in detail how the dependent variable is constructed and its rationale, including the specific indicators selected, the measurement method, and the rationale of the score. In addition, the theoretical basis for using this variable is also discussed in the newly added section above (i.e., the section added in response to the first reviewer's comment). The relevant parts have been marked in red for easy review. We hope that these additions will make the measurement method of this article clearer and more convincing.

5.Please explain the rationale for the choice of instrumental variables

Thank you for the reviewer's valuable comments. We have discussed in detail the construction method and reasons for using the instrumental variables, and revised the relevant parts. The main thing is that the instrumental variable can meet the requirements of relevance and exogeneity to ensure its eligibility. I hope these additions can make the selection of instrumental variables in this article more reasonable and convincing.

6.References in the text are not formatted correctly, please refer to the journal formatting

It has been modified according to the journal format.

Reviewer #2: authors should provide revisions that strengthen the theoretical foundation, expand the literature review, enhance methodological rigor, ensure data representativeness, employ rigorous data analysis, offer an in-depth discussion, address potential endogeneity issues。

Thank you for your valuable comments. Based on your suggestions, we have comprehensively sorted out and discussed the article's introduction, literature review, theoretical assumptions, solutions to endogeneity problems, and variable settings. The specific changes are as follows: Strengthen the theoretical basis: Add more relevant literature and theoretical support to the introduction and literature review to strengthen the theoretical basis of the article; Expand the literature review: Expand the literature review section and cite more recent research results to provide more comprehensive background information; Improve the rigor of the method: Detailed explanation of the selection of variables and data processing methods to ensure the rigor of the method; Ensure data representativeness: Detailed discussion of the representativeness of the data to ensure that the data sample is representative; Adopt rigorous data analysis: Adopt more rigorous data analysis methods and describe the analysis process in detail. Solve potential endogeneity problems: Describe in detail the reasons for selecting instrumental variables and how they are constructed to ensure the rationality of the selection, and in-depth explanation of other robustness test methods. The relevant parts have been marked in red.

Reviewer #3:

The following comments are listed in the order of the paper’s development.

(1) Both the motivation and the contribution of the paper are weak. The paper motived this research by China’s common prosperity policy. This means that the research has little policy implications for a wider research community. China’s common prosperity policy is only the observed problem for research, which should be raised to the academic level by formulating a research question that can be located to a literature.

Thank you for the valuable comments from the reviewers. We have reorganized the introduction and the Theoretical Analysis and Research Hypotheses section of the article, and elaborated on the motivation and contribution of the article in more detail. We have expanded the impact of economic policy uncertainty on the common prosperity of enterprises to a more general academic level to enhance the breadth of research and policy influence. The relevant modified parts have been marked in red for easy review. We hope that these changes can enhance the academic value and contribution of this article.

(2) Related to the above comment, the paper subjectively linked EPU with IECP without properly discussing the underlying theory to justify this relationship. Although the literature section contains some studies, it is unclear what the underlying economic or finance theory is.

Thank you for your valuable comments. We have re-examined the theoretical assumptions on the impact of economic policy uncertainty (EPU) on intra-enterprise common prosperity (IECP) and clarified the logical relationship and theoretical basis of the relationship between EPU and IECP. We have discussed the relevant economic and financial theories in detail in the article to support the rationality and academic nature of this relationship. The relevant revisions have been marked in red for easy review.

(3) The paper needs to explicitly justify the definition and construction of the IECP score for the firm. What is the economic meaning of the constructed IECP?

Thank you for the reviewer's valuable comments. We have explained in detail the definition and rationality of the construction of the company's IECP score, including the specific construction method and theoretical basis. In addition, we discussed the economic significance of IECP in the newly added subsection 2.1 "The connotation of common prosperity within the enterprise". The relevant modified parts have been marked in red for easy review. We hope that these additions will make the measurement method and economic significance of this article clearer and more convincing.

(4) In Section 4.2.1, the paper wanted to exclude the effect of the stock market volatility, hence the paper simply deleted the 2015 observations for the sample firms to eliminate the impact of China’s 2015 stock market volatility. Such a treatment implies that the data used in the estimation is no longer a properly panel data set.

Thank you for your valuable comments. We deleted the 2015 data in the robustness test in order to verify the adaptability of the data in different situations. After deleting the 2015 data, the conclusion of the article did not change, which just shows that our data conclusion is robust and reliable and can be used for subsequent research. Therefore, this treatment does not affect the appropriateness of the data set, but enhances the credibility of the conclusion. I hope these explanations can clarify your doubts. Thank you again for your suggestions.

(5) In Section 4.3., the paper used ‘the uncertainty of US economic policy’ as an instrument for China’s EPU, but the paper did not explain what ‘the uncertainty of US economic policy’ means, how this variable is defined/constructed, and where the data on this variable is taken from. More importantly, the paper did not even explain why ‘the uncertainty of US economic policy’ is suitable to be an instrumental variable for China’s EPU.

Thank you for the reviewer's valuable comments. We have already described in detail the definition, construction method and data source of the instrumental variable "U.S. economic policy uncertainty" in the original paper. In addition, we have also explained in detail why "U.S. economic policy uncertainty" is suitable as an instrumental variable for China's EPU. The relevant parts have been marked in red for easy review. I hope these additions can make the choice of instrumental variables in this article clearer and more convincing.

(6) In Section 4.4., the paper undertook a PSM test. However, the motivation of this test is unclear. The explanation about how the paper did the PSM is also unclear. For example, the way in which the paper defines the treatment group and control group is very confusing.

Thank you for the valuable comments from the reviewers. Considering the refinement and usefulness of the article, we carefully evaluated the contribution of the PSM test to the robustness test of the article and decided to delete the relevant parts. This can remove redundant content that contributes little to the article and enhance the refinement and readability of the article. We hope that these modifications can improve the overall quality of this article.

(7) In Section 5.1, the paper did not explain how TFP is constructed. A method is mentioned without the explanation how it is constructed.

Thank you for the reviewer's valuable comments. We have described in detail how TFP (total factor productivity) is constructed in Section 5.1 and marked the relevant parts in red for easier review. We hope that these additions will make the measurement method of this paper clearer and more convincing.

(8) The result in column (2) of Table 7 is confusing. In column (2) the estimated coefficient for lnEPU is positively significant in explaining IECP, and the estimated coefficient for the interactive term between lnEPU and productivity (lnlp) is negatively significant in explaining IECP, putting together this result suggests that higher productivity reduces the positive effect of EPU on IECP, which is in the opposite to what the paper wants to prove.

Thanks to the reviewers for their valuable comments. We have explained in detail how productivity is built and conducted an in-depth analysis of the moderating effects. Based on the basic regression, we gradually added moderator variables and interaction terms.

In column (1), we add total factor productivity (TFP) and find that it significantly changes the sign of the explanatory variable (from negative to positive), which indicates that TFP may be an important mechanism variable that can improve economic policy uncertainty. The negative relationship between sex (EPU) and intra-firm shared prosperity (IECP). Therefore, in column (2), we add the interaction term of the explanatory variable and the moderator variable, and the results show that it significantly alleviates the negative relationship in the base regression, which is consistent with our expectations.

Regarding the change in sign of the explanatory variables, the addition of moderator variables and interaction terms requires re-estimation of the negative effects of the original basic regression. The added model already considers the impact of the moderator variable on the explanatory variable at different levels, so in a model with moderator variables, the estimated value of the explanatory variable does not need to be considered too much. The results of the interaction term in this article have demonstrated the establishment of the core hypothesis.

Hopefully these explanations will clear up your doubts. Thanks again for your advice.

(9) In Table 8, the paper tested the difference in the result between SOEs and non-SOEs. But the paper did not explain how SOEs is defined. Moreover, the paper compared the results between the two groups based on the raw estimates, which is wrong (the same comment applies to both Table 10 and Table 11). In addition, according to the results in Table 8, the IECP of non-SOEs is less affected by EPU, while the negative impact of EPU on the IECP of SOEs is more profound. This result is not consistent with the Chinese practice. The impact of EPU on Chinese non-SOEs should be more profound as compared to that of Chinese SOEs.

Thank you for the reviewer's valuable comments. We have explained the definition of state-owned enterprises in detail in the article. In addition, considering the drawbacks of comparing the two groups of results directly based on the original estimates, we used the inter-group difference coefficient to compensate for it on this basis to ensure that the differences between the groups can be accurately compared.

Regarding the reviewer's point that the impact of EPU on China's non-state-owned enterprises should be more far-reaching, we believe that state-owned enterprises have a stronger policy orientation than non-state-owned enterprises. When the country's macroeconomic policies change, state-owned enterprises have less flexibility and can only accept government regulation more directly, which also determines that the uncertainty of economic policy changes will have a greater impact on state-owned enterprises. Therefore, it is reasonable that our results show that the negative impact of EPU on the IECP of state-owned enterprises is more far-reaching.

I hope these explanations can clarify your doubts.
