# Supplementary material for: Economic policy uncertainty and common prosperity within the enterprise: Evidence from the Chinese market
Source: PLoS One. 2024 Oct 21;19(10):e0309370. doi: 10.1371/journal.pone.0309370 (PMC11493295; doi:10.1371/journal.pone.0309370)
Supplement: Attachment — Submitted filename: Response to review comments.docx [file pone.0309370.s004.docx]

First of all, I would like to thank the reviewers and editors for their valuable suggestions. We have improved the four areas you mentioned, taking into account clarity, broad applicability, and limitations. The following is my response to the review comments.

**Clarity and Structure:**

1、Please revise the introduction (Section 1) to provide clearer contextualization of the study's objectives and the significance of the research question addressed. Consider succinctly summarizing the theoretical framework and key hypotheses.

Thank you for the reviewer's valuable comments. We have reorganized the introduction, clarified the research objectives, and highlighted the importance of the research questions. In addition, in Section 2, "Theoretical Analysis and Research Hypotheses", we briefly summarize the theoretical framework and key hypotheses through a conceptual framework diagram. The relevant parts have been marked in red.

2、In Section 3.2.1 (Dependent Variable), ensure that the definition and operationalization of "common prosperity" are clearly articulated. Specify the exact methodology used for constructing the enterprise common prosperity score.

This article has clarified the definition and construction method of common prosperity, and announced the details of the selection of three-level indicator variables. The variable construction method of this article is also clear, mainly reverse indicator adjustment, missing value filling, standardization and weighting to obtain the common prosperity score within the enterprise, and finally divide it into 9 levels. The relevant parts are marked in red.

3、Improve the flow of the methodology section (Section 4.3) by clearly delineating between the first and second stages of the instrumental variable regression. Additionally, provide a more detailed explanation of the rationale behind choosing US economic policy uncertainty as the instrumental variable.

This paper clearly divides the first and second stages of instrumental variable regression and improves the method flow. In addition, this paper elaborates on the reasons for selecting US economic policy uncertainty as an instrumental variable from the perspective of correlation and exogeneity. The relevant parts are marked in red.

**Contextualization and Generalizability:**

4、Expand the discussion in Section 5 on the implications of your findings beyond the specific context of listed companies in China. Discuss how the results might apply to other sectors or countries facing similar issues of economic policy uncertainty and corporate social responsibility.

Thanks to the reviewers for their valuable comments. We have expanded the discussion in Section 5 to explore the applicability of economic policy uncertainty, corporate social responsibility, total factor productivity and internal prosperity in different industries and countries from an international and industry perspective. The relevant parts are marked in red.

5、Consider adding a subsection under Section 5.2.1 (Equity Nature Perspective) to explicitly discuss the potential limitations and biases associated with using state-owned enterprises (SOEs) and non-state-owned enterprises (non-SOEs) as distinct groups. Address the generalizability of findings to different organizational structures.

Thank you for the reviewer's valuable comments. We have discussed the generalizability of different organizational structures in Section 5.2.1 and added a new Section 5.2.2 "Potential limitations and biases" to discuss in detail the limitations and biases of using state-owned enterprises (SOEs) and non-SOEs as different groups. The relevant parts have been marked in red.

**Discussion of Limitations:**

6、Provide a more comprehensive discussion of potential limitations associated with the data sources (e.g., CSMAR) and the methodology used (e.g., IV regression). Discuss how these limitations might affect the interpretation of your results and suggest avenues for future research to address these limitations.

Thank you for your valuable comments. We have added a new section 6.2 "Discussion of Limitations" under the conclusion of Section 6, which elaborates on the potential limitations of the article from the two aspects of data limitations and method limitations, and proposes solutions and directions to these limitations in section 6.2.3 "Future Research Directions". The specific changes have been marked in red in the original text. I hope these changes can better meet the requirements of the reviewers and improve the overall quality of the paper. Thank you again for the reviewers' valuable suggestions.

**Interpretation of Results:**

7、Strengthen the discussion in Section 5.1 (Moderation Effect Test) by offering deeper insights into the practical implications of your findings for policymakers and practitioners interested in promoting common prosperity within enterprises.

Thanks to the reviewers’ comments, we have strengthened the discussion in Section 5.1.
